# A hierarchical model of transcriptional dynamics allows robust estimation of transcription rates in populations of single cells with variable gene copy number

**DOI:** 10.1093/bioinformatics/btt201

**Published:** 2013-05-14

**Authors:** Dan J. Woodcock, Keith W. Vance, Michał Komorowski, Georgy Koentges, Bärbel Finkenstädt, David A. Rand

**Affiliations:** ^1^Warwick Systems Biology Centre and ^2^Department of Life Sciences, University of Warwick, Coventry, CV4 7AL, ^3^MRC Functional Genomics Unit, Department of Physiology, Anatomy and Genetics, University of Oxford, South Parks Road, Oxford, OX1 3PT, ^4^Department of Statistics, University of Warwick, Coventry, CV4 7AL, UK and ^5^Institute of Fundamental Technological Research, Polish Academy of Sciences, Pawińskiego 5B, 02-106 Warszawa, Poland

## Abstract

**Motivation:** *cis*-regulatory DNA sequence elements, such as enhancers and silencers, function to control the spatial and temporal expression of their target genes. Although the overall levels of gene expression in large cell populations seem to be precisely controlled, transcription of individual genes in single cells is extremely variable in real time. It is, therefore, important to understand how these *cis*-regulatory elements function to dynamically control transcription at single-cell resolution. Recently, statistical methods have been proposed to back calculate the rates involved in mRNA transcription using parameter estimation of a mathematical model of transcription and translation. However, a major complication in these approaches is that some of the parameters, particularly those corresponding to the gene copy number and transcription rate, cannot be distinguished; therefore, these methods cannot be used when the copy number is unknown.

**Results:** Here, we develop a hierarchical Bayesian model to estimate biokinetic parameters from live cell enhancer–promoter reporter measurements performed on a population of single cells. This allows us to investigate transcriptional dynamics when the copy number is variable across the population. We validate our method using synthetic data and then apply it to quantify the function of two known developmental enhancers in real time and in single cells.

**Availability:** Supporting information is submitted with the article.

**Contact:** d.j.woodcock@warwick.ac.uk

**Supplementary information:** Supplementary data are available at *Bioinformatics* online.

## 1 INTRODUCTION

The rate of transcription of RNA polymerase II transcribed genes is determined by interactions between general transcription factors assembled at the core promoter and sequence-specific transcription factors bound to *cis*-regulatory DNA sequences, such as enhancers. Experiments in cell populations have suggested that enhancers function either as rheostats, by increasing the rate of transcription initiation from a promoter in a graded manner, or as on/off switches increasing the proportion of cells transcribing a gene without affecting the rate (Jeziorska *et al.*, 2009). However, recent studies have shown that even though gene expression levels seem to be precise when averaged over a large population of cells, the process of transcription in individual cells is stochastic (Elowitz *et al.*, 2002; Paulsson, 2005). A mammalian gene has intermittent random bursts of expression in a single cell separated by refractory periods of inactivity with the kinetics of this process varying widely between genes (Harper *et al.*, 2011). This results in variability in protein expression both within individual cells and between cells in a population (Paulsson, 2004). Although the kinetics of transcription has been studied in single cells, the ability of enhancers to regulate transcription at single-cell resolution remains poorly understood.

Studies with stable cell lines containing integrated luminescent and fluorescent reporters have been used to measure fine-scale dynamics of transcription (Harper *et al.*, 2011; Suter *et al.*, 2011). This approach could, in theory, be used for large-scale analyses of enhancer function, but transient transfection is more amenable because of the numbers of constructs involved. However, transient transfection has a major disadvantage in that the variation in copy number makes the reliability of any quantification problematic. It is, therefore, of considerable interest to provide a method that can deal with copy number variation and estimate transcription rates using transient transfection. To address this problem, we have developed a hierarchical Bayesian model to estimate transcriptional dynamics in single cells, and we have used it to gain a more detailed understanding of *cis*-regulatory enhancer function.

Our hierarchical model builds on previous models of gene transcription (Finkenstädt *et al.*, 2008) and uses the linear noise approximation (Elf and Ehrenberg, 2003) to establish a likelihood function that enables us to estimate the model parameters using Markov Chain Monte Carlo (MCMC) (Komorowski *et al.*, 2009). This forms the first ‘layer’ of the hierarchical structure of the model and incorporates the variation within an individual cell. The second layer models the variation between cells. This has a dual function: it provides information about extrinsic noise and heterogeneity (Elowitz *et al.*, 2002) that is of considerable value in itself, and, importantly, it aids the estimation process making it more robust. In this approach, we assume that some of the parameters for each cell are drawn from an overarching distribution at the population level. By estimating the parameters of these distributions (henceforth referred to as hierarchical distributions) alongside the individual cell parameters, we can gain information about the entire population of cells. This allows a much more principled and informative method of estimating these distributions than is achieved by treating the single cells separately and then subsequently pooling the statistics to get population estimates. As the inference of the hierarchical distributions is performed concurrently with the estimates for the single cells, the parameter estimation procedure can be carried out in such a way that the single-cell parameters inform the hierarchical population distribution, which in turn provides information for the individual cell estimates.

This cyclical information transfer, sometimes referred to as *borrowing strength* from the other parameters, not only allows us to estimate parameter distributions but also enables us to extract information about parameters that may not previously have been available. Such a situation arises here, where we try to estimate transcription rates from reporter protein measurements using a model of protein and mRNA kinetics. The problem is that for a single cell, the production term for the mRNA is proportional to the product of the single-copy transcription rate 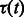 and the gene copy number *c* for that cell. These two values are then inseparable and thus unidentifiable. However, with the hierarchical model, robust quantitative analysis can still be performed when the copy number is allowed to vary, although with the caveat that we cannot identify absolute values for the per-copy transcription rate. Despite this, we can estimate the *ratio* of the per-copy transcription rate between given promoter structures and can, therefore, deduce the function of the individual *cis*-regulatory elements. We apply this algorithm to both simulated and experimental data. The former allows us to test the effectiveness and reliability of the algorithms at reconstructing the statistics of the known underlying process, and the latter shows that these techniques can provide informative insights into the kinetics of real regulatory elements that would not be possible with bulk-cell methods.

## 2 METHODS

### 2.1 Mathematical model of gene expression

We follow the conventional model of gene expression (Paulsson, 2005) in which a gene transcribes mRNA, which is subsequently translated into protein. The protein in question is assumed to be a reporter protein that can be detected by a microscope. We assume that the molecule numbers are sufficiently high; therefore, we can model the creation and degradation of mRNA and protein as a continuous stochastic process (Finkenstädt *et al.*, 2008) and, hence, model the system as a pair of stochastic differential equations
(1)
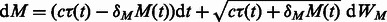

(2)
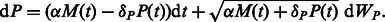

We also model the microscope detection of the fluorescence in a measurement equation
(3)
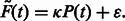

Equation (1) describes the change in mRNA concentration in a time of duration 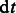 in a cell containing *c* plasmids where each plasmid transcribes mRNA at a rate according to 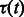. The mRNA in the cell, *M*(*t*), degrades at rate 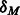. Similarly, Equation (2) describes the change in protein concentration in time 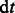. Here, protein is translated at rate α, dependent on the mRNA concentration *M*(*t*) and is degraded at a rate 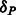 proportional to the protein concentration *P*(*t*). The terms in the square root represent the noise expected in the process, which arises as a result of the Central Limit Theorem applied to the number of events in the birth/death process (Heron *et al.*, 2007), and 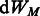 and 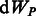 represent Wiener processes that model the intrinsic stochastic fluctuations of the processes. In the measurement equation [Equation (3)], κ is the fluorescence per mole of protein, and ε is an additive measurement error term taken from the distribution 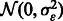.

There is evidence that transcription can occur in a number of ways, from short pulses to sustained bursts and with stalling and other refractory mechanisms involved (Ingram *et al.*, 2008). In these cases, any information about the transcriptional mechanism would have to be encoded in the transcription function 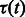. For clarity and simplicity, here we will assume a simple changepoint functional form in which transcription may occur at two levels: a low level, corresponding to basal transcription levels (an *off-phase*), which subsequently leads to a high level where active transcription is taking place (an *on-phase*). We also assume that the plasmid copies switch from the off-phase to the on-phase at the same time. Thus, we assume that

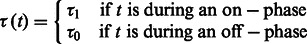

In this study, we only assume that there is one transition between the two states, from the off-phase to the on-phase. As such, this form of the transcriptional model also has the advantage of a parsimonious parameterization, as it only requires three parameters: the two values of τ that correspond to the active and inactive phases, and a time *s* when the changepoint, henceforth referred to as a *switch*, occurs.

### 2.2 Hierarchical Bayesian model

As the total transcription rate in Equation (1) is given by 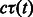, the parameters *c*, 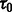 and 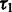 are not identifiable, and the most that we can hope to estimate is 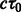 and 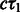. In fact, we shall not attempt to evaluate absolute values of 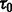 and 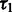 but shall instead be interested in comparing the relative rates corresponding with two or more promoter constructs. If, for example, we make the unreasonable assumption that the copy number *c* is the same for these constructs in all cells, then if we can estimate 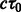 and 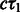 for each construct, we can evaluate the ratios of the transcription rates between them and thus determine the extent to which they enhance or repress transcription.

We do not make this assumption but instead note that it is reasonable to assume that the variation of the copy number can be modelled by a common probability distribution across all cells. In fact, if we constrain *c* so that it is drawn from a common distribution, then we can decouple the two parameters in a similar way to the above case where *c* was constant. This is because each *c* value will be estimated with respect to the rest of the *c* values in the population via the distribution; therefore, the potential values that would be viable as an estimate are restricted. Therefore, it follows that if we estimate values of 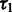 and 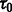 for each promoter construct, then these transcription rate estimates will also be restricted, as they are contingent on values of *c*, which are themselves constrained by their common distribution. This means that the relative transcription rates for each construct will be comparable at the population level, as the estimates are all dependent on the same underlying distribution over *c*. Equally, the converse is true; therefore, if we assume that the transcription rates for each construct are also drawn from a common distribution, then the estimates of *c* will be constrained by the distributions over the transcription rates. As such, by assuming distributions over the transcription rates and the copy number, the estimates will borrow strength from each other, and this will further facilitate the identification of the parameters.

Furthermore, if we similarly assume a probability distribution over some of the other parameters, this will assist in the decoupling of the various rates. In the model described in Section 2.1, we would expect that the values of α and 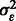 would also be similar between cells and warrant modelling with a distribution. We also assume that the variation in κ would be negligible and make it equal for all cells. Conversely, for the purposes of this investigation, we would expect that the switch times will be independent and, hence, will not be amenable for modelling with a distribution. We can now construct a hierarchical Bayesian model, reflecting these assumptions, which will allow the estimation of these distributions alongside the single-cell parameters.

Given data 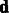 and parameters 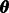, a non-hierarchical Bayesian analysis starts with a prior distribution 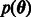 and likelihood 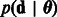 and uses these to compute a posterior probability 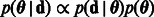. In our case, 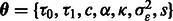. Using a hierarchical model, we treat a group of time-series data 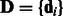 coming from single cells in a common framework. We estimate the parameter values 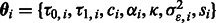 for each time-series 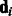. We divide 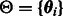 into those parameters that will be modelled with the hierarchical approach, 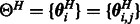 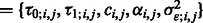 and those that are not, 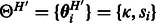.

We then introduce new parameters 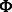 to describe a probability distribution 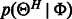 on 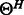 and replace the prior 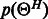 by the prior 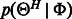. Together with a hyperprior 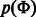, this results in a posterior probability

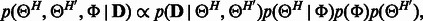

where, for *n* cells and *m* hierarchical distributions,

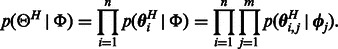



We assume that each of the 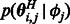, except those corresponding to the variance of the measurement error and copy number, is lognormal distributions where 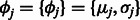, and μ and σ are the mean and standard deviation. For the variance of the measurement error, we assume a gamma distribution over 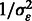, as this is the standard prior for the precision of a normal distribution in a hierarchical framework. Finally, we assume a truncated Poisson distribution (David and Johnson, 1952) for the copy number as, in transient transfections, a plasmid entering a cell can be considered as an event, and the Poisson distribution is the correct way to describe a count of independent events in a time interval. This distribution is truncated at zero as if no plasmids enter the cell then we will be unable to detect them and include them in the analysis. As the magnitude of the transcription rates and the copy number is indistinguishable, we use a continuous form (Marsaglia, 1986) to calculate the pdf of the Poisson distribution, in which the factorial is replaced by a gamma function and is defined as
(4)
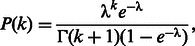

where 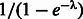 is the renormalization term included to account for the truncation at zero.

Using this framework, we can estimate the transcription rates of each cell conditional on the other cells containing the same construct via the corresponding hierarchical distribution. As these rates are estimated relative to the copy number distribution, which is the same across all cells regardless of construct, these distributions are comparable with each other. It should be noted that in the absence of a suitable control population, it is not possible to determine the exact copy number or transcription rates as they are only defined with respect to the other. As such, comparisons can only be made in terms of the relative differences between the constructs.

### 2.3 Parameter estimation

**Fig. 1.**
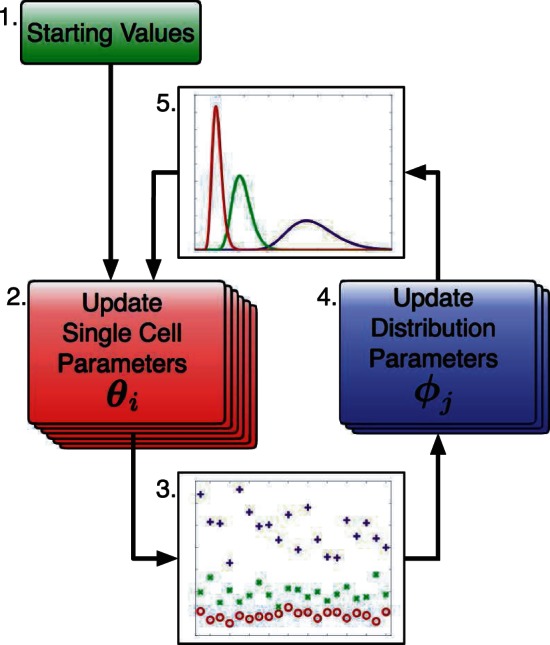
A schematic diagram highlighting the flow of information through the hierarchical estimation procedure. The starting values (1) for each cell are updated using the likelihood derived from their single-cell time courses (2). These estimates (3) are then used to update the parameters of the hierarchical distributions over the single-cell parameters (4). The distributions (5) are then used to inform the next set of single-cell estimates (2). This process is repeated until both sets of parameters have converged to a stationary distribution

We use Metropolis–Hastings MCMC to estimate the parameters (conditions given in the Supplementary Data). A schematic representation of the algorithm is given in Figure 1. The likelihood for the individual gene expression model for each cell was calculated using the linear noise approximation (LNA) (Elf and Ehrenberg, 2003; Komorowski *et al.*, 2009). Although the formulation of the LNA requires the assumption of high-molecule numbers, empirical evaluation has shown that the LNA approximation remains valid for low numbers of mRNA (5–35) and protein (100–500) molecules (Komorowski *et al.*, 2009). As all the parameters are positive, we sampled the logarithms of the parameters and corrected the posterior estimate with the Jacobian. As we sample in log-space, it is natural to estimate the parameters of the normal distribution underlying each lognormal hierarchical distribution directly. For the measurement error variance and copy number distributions, we converted back from log-mean and variance estimates to the relevant parameters. As we are estimating parameters for all the cells together, the algorithm can be slow; therefore, we used a parallelized block-updating algorithm in which the number of cells to be updated in each iteration was chosen to be equal to the number of processor cores available. In the time-series parameter estimation step, the calculation was split so that on each core we proposed three new parameters for each of the chosen cells based on a normally distributed perturbation from the old parameter value, calculated the log-likelihoods using the LNA and then returned the likelihood values to the main program, which summed them and accepted or rejected in the usual Metropolis–Hastings fashion. Aside from a speed increase proportional to the number of cores available, this method also has the advantage of better mixing and fewer correlations over the standard Metropolis–Hastings algorithm. This also means the algorithm will scale to much larger datasets if a sufficiently large cluster computer is available. The hierarchical parameters were subsequently updated in serial in the standard manner. Another implementation issue to note is that the normalization constant should not be omitted when calculating the individual cell likelihoods in the MCMC procedure. This is because the time series may be of different lengths, and the omission of the normalization constant in the LNA will result in long time series having a disproportionately greater effect on the combined likelihood than short time series.

## 3 RESULTS

### 3.1 Synthetic data

Three datasets, Group A, Group B and Group C, of synthetic data were generated using the Gillespie algorithm based on the model given in Section 2.1. The transcription and translation rates were drawn from lognormal distributions, the measurement error variance drawn from an inverse gamma distribution and the copy number drawn from a Poisson distribution. Group A consisted of cells with a low-active transcription rate mean (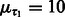), Group B consisted with a medium-active transcription rate mean (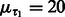) and Group C consisted of cells with a high-transcription rate mean (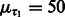). The variances of the transcription rates were assumed to be the same as the mean; hence, the Fano factor was always equal to 1. The other parameters were drawn from the same distributions for all groups, the values of which can be found in the Supplementary Data. There was only one switch from inactive to active gene transcription; therefore, there will be little information on the degradation rates. These were assumed to be known and were fixed at the correct values for the estimation.

To evaluate the effectiveness of the hierarchical model, we ran the algorithm once with the standard non-hierarchical likelihood (model S) and once with the full hierarchical likelihood (model H). In the algorithm using the standard model, we used uninformative priors over the parameters, and the mean and variance reported in this case were calculated at the end of the estimation procedure using the means of the chains for each cell. In the algorithm using the hierarchical model, we updated the hierarchical distributions alongside the regular parameter updates, and the mean and variance reported are calculated from the distributions constructed using the mean of the chains for the hierarchical parameters. As we are primarily interested in comparisons between the groups, we only report the ratios between the active transcription rates of the three groups; the full parameter estimates and a discussion on their accuracy can be found in the Supplementary Data. It should also be noted that differing numbers in each group does not adversely affect the estimation (see Supplementary Data), and in this case, we chose equal numbers solely to facilitate the subsequent comparison.

The ratios between the three groups, given in Table 1, show that the parameter estimates performed using the hierarchical procedure are significantly more robust than the standard procedure in reproducing the magnitude of the difference between the groups. Furthermore, another hierarchical estimation run was performed on synthetic data created with the same transcription rate distributions but using a higher mean copy number, which returned similar results, indicating that the value of the copy number has no effect on the ability of the algorithm to reproduce these ratios (see Supplementary Data). We can investigate why this is so by examining the aggregate behaviour of the individual transcription rate estimates that inform the hierarchical distributions.

**Table 1.** Ratios of mean transcription rate estimates between Groups A, B and C

Estimation Method	B/A	C/B	C/A
Actual ratio	2	2.5	5
Standard ratio	2.94	3.29	9.71
Hierarchical ratio	2.11	2.49	5.25

**Fig. 2.**
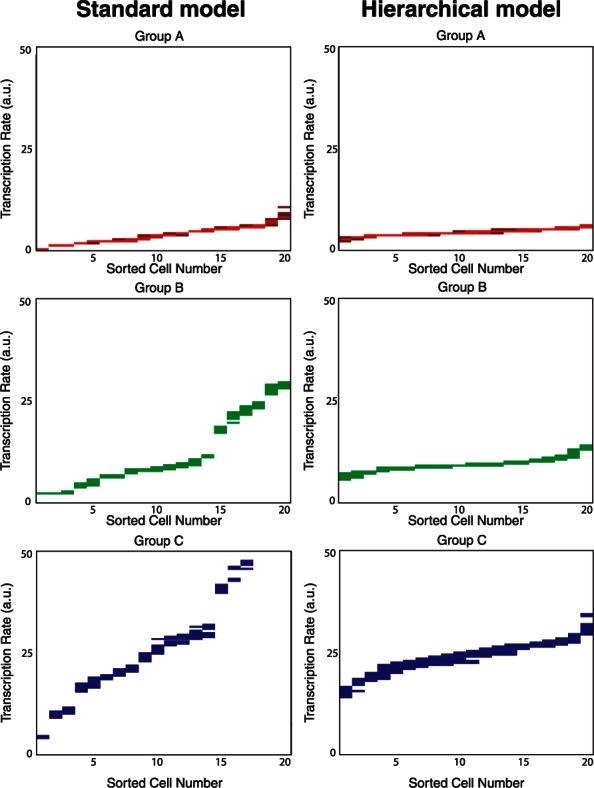
Comparison of the relative transcription rate estimates of synthetic data Groups (**A**) (top), (**B**) (middle) and (**C**) (bottom) for the standard non-hierarchical model (left) and the full hierarchical model (right). The coloured bars represent the distribution of the Markov chain estimates for that cell in which a high-probability mass corresponds to a light colour ranging to a dark colour for low-probability mass. All units are arbitrary

Figure 2 shows the transcription rate estimates sorted into ascending order for each group for both the standard and the hierarchical estimation procedures. These values should not be considered as an accurate transcription rate estimate for each cell because there is still some ambiguity in the estimate at the individual cell level, as the exact copy number is unknown. However, as they are all estimated relative to the same copy number distribution, we can use information from the collective behaviour of the individual estimates. We can immediately observe that the three distinct parameter ranges are distinguishable for each group in both procedures, but there is a larger range of values in the estimates using the standard model than the hierarchical one. Also, although these are on comparable scales, the range of estimates for each group is much tighter in the hierarchical than in the standard procedure. Furthermore, the actual distributions of the MCMC estimates are often much tighter when the hierarchical model is used, as the estimates are more likely to spread over a wider range of values. These observations highlight the adverse effect the trade-off between the copy number and transcription rate values can have on the estimations, and how using the hierarchical model overcomes this.

### 3.2 Real data

We applied the method to investigate how different enhancer regions affect the way transcription rates are distributed in a population of living cells.

**Fig. 3.**
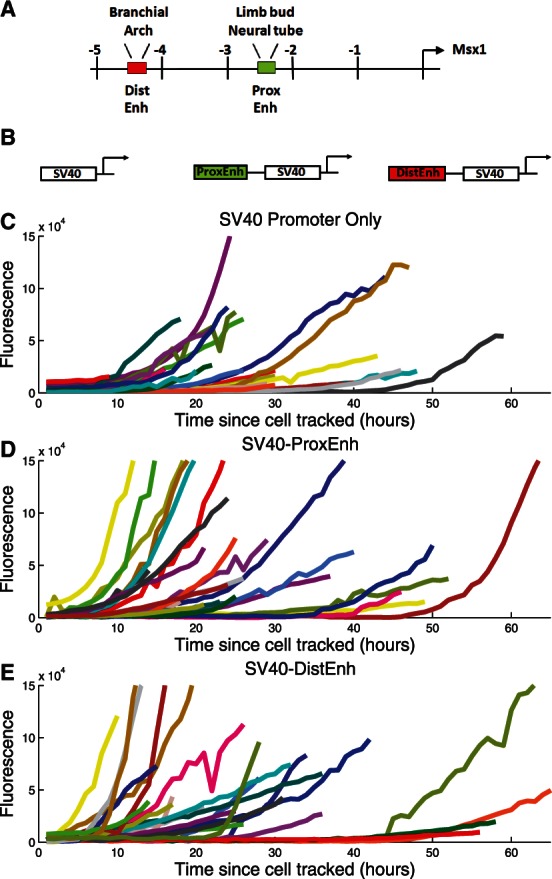
Generation of the datasets. Pane (**A**) shows a schematic diagram showing the locations of the two enhancers respective to the transcription start site in the Msx1 gene, with (**B**) showing the three corresponding reporter protein constructs. The three lower panes show onset curves from cells containing (**C**) the promoter only, (**D**) the proximal enhancer and (**E**) the distal enhancer

The Msx1 transcription factor is expressed in mesenchymal precursor cells at multiple locations in the developing mouse embryo. Two enhancer regions have been shown to control Msx1 expression (Fig. 3A). The proximal enhancer (ProxEnh) situated 2.2 kb upstream of the Msx1 TSS activates expression in the first branchial arch and dorsal neural tube, whereas the distal enhancer (DistEnh) at 4.0 kb upstream upregulates Msx1 expression in the limb mesenchyme, second branchial arch and the myotome (MacKenzie *et al.*, 1997).

C2C12 myoblasts, derived from mouse satellite cells, have previously been used to study Msx1 transcriptional control. Msx1 is expressed in proliferating C2C12 myoblasts but not in differentiated C2C12 myotubes while mis-expression of Msx1 in differentiated C2C12 cells induces the dedifferentiation of myotubes into multiple mesenchymal progenitors (Odelberg *et al.*, 2000). To study Msx1 enhancer function, we first tested whether the known Msx1 enhancers are active in C2C12 myoblasts. To do this, the Msx1 proximal and distal enhancers were cloned upstream of the heterologous Simian vacuolating virus 40 (SV40) promoter in the pGL3 luciferase reporter (Fig. 3B), and the activity of these constructs compared with the SV40 promoter alone in a transient transfection assay. The results of this experiment are given in the Supplementary Data and reveal that the ProxEnh and DistEnh containing reporters are 4.2-fold and 4.9-fold more active compared with the SV40 promoter alone. Transient transfection of enhancer–promoter reporters in C2C12 cells, therefore, represents a good system to study Msx1 enhancer function in populations of individual cells.

We next replaced the luciferase gene with a nuclear localized variant of the gene encoding the Venus fluorescent protein (Jeziorska *et al.*, 2012) to generate SV40, ProxEnh-SV40 and DistEnh-SV40 Venus reporters (Fig. 3). These constructs were transiently transfected into C2C12 cells (experimental methodology can be found in the Supplementary Data) and analysed using single-cell time-lapse microscopy in combination with custom tracking and segmentation algorithms to generate fluorescent time courses for each construct (Downey *et al.*, 2011). From these, we randomly selected 25 cells for each construct and assembled fluorescent onset curves from the time of transfection to the point when maximal fluorescence was reached. These datasets are shown in Figure 3C–E.

We calculated transcription rate estimates for all 75 single-cell fluorescent reporter onset curves simultaneously using the hierarchical Bayesian model as outlined in Section 2. The algorithm is robust to choices of the degradation rate parameters, as the transcription rate information is contained in the ascending part of the onset curves (Fig. 3C–E). This is because the rate of increase in mRNA and subsequently reporter protein levels caused by the higher levels of transcription by far outweighs the rate at which those molecules degrade, particularly as the Venus reporter used in these experiments is highly stable. As such, degradation rate parameters were fixed to values estimated from population experiments (see Supplementary Data) and the transcription rate estimations conditioned on these parameters providing a consistent basis for comparison. The mean values generated from this were then used as the parameter estimates.

The estimated mean, variance and coefficient of variation of the hierarchical distributions are given in Table 2. These clearly show that the enhancer function of the two Msx1 regulatory elements is recovered using the model as the presence of either the ProxEnh or DistEnh increases the mean transcription rate. Although the mean of the proximal enhancer is approximately the same as that of the distal enhancer, the coefficient of variation is higher in the distal enhancer, indicating that the extrinsic noise in the population increases at least partially independently of the transcription rate.

**Table 2.** Population-level relative transcription rate mean, standard deviation and coefficient of variation estimated for each promoter construct

Group	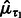	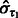	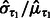	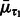	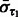	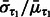
Promoter only	34.31	19.86	0.58	33.66	16.32	0.48
Proximal enhancer	44.34	35.73	0.80	43.28	29.79	0.68
Distal enhancer	44.30	49.99	1.12	44.07	37.52	0.85

*Note*: The 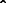 above the statistic denotes those obtained directly from the hierarchical distribution, and the 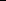 above the statistic denotes that the population statistics are calculated from the mean values of the individual MCMC estimate. These values are conditional on the common copy number distribution and do not represent the absolute transcription rates. All units are arbitrary.

**Fig. 4.**
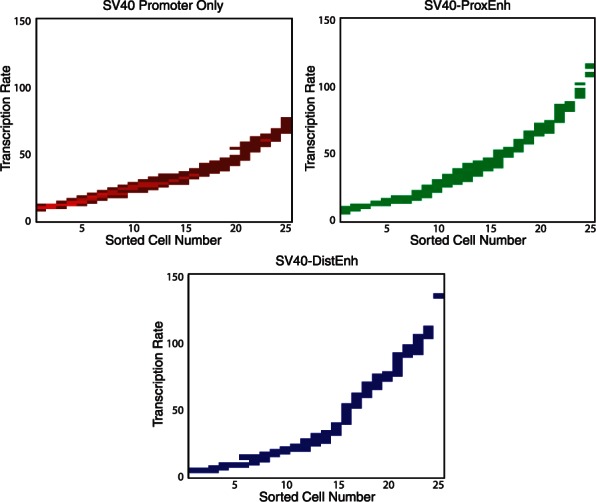
Comparison of the transcription rate estimates of for cells containing the promoter only (top left), the proximal enhancer (top right) and the distal enhancer (bottom) estimated using the full hierarchical model. The coloured bars represent the distribution of the Markov chain estimates for that cell in which a high-probability mass corresponds to a light colour ranging to a dark colour for low-probability mass

Moreover, we can also investigate the contribution of each individual cell to the relative transcription rate distribution by analysing the first layer estimates corresponding to each cell. Figure 4 shows the transcription rate estimates generated from the MCMC chains for each construct sorted into ascending order by their means. We observe that the range of transcription rates in individual cells containing the ProxEnh and DistEnh constructs is greater than that obtained by the promoter alone, consistent with the results in Table 2. In addition, the results show that although the maximum transcription rate achieved in cells containing the SV40 promoter alone is substantially lower than those in the ProxEnh and DistEnh groups, ∼60% of the cells containing the enhancers transcribe at similar rates to the cells with the promoter only. This is important, as it implies that enhancers only have an effect on a proportion of the cellular population rather than providing an incremental increase to the entire population.

## 4 DISCUSSION

We have presented a method of extracting comparable transcription rates from populations of single cells with variable copy number and validated it on synthetic datasets. Previously, all single-cell analysis would have been performed on a population of cells with a known copy number, as this unknown variable renders any robust analysis of transcription intractable. With our method, constructed under the assumption that the rates involved in transcription are drawn from a statistical distribution, we can decouple the processes involved in transcription, allowing the estimation of values relative to each other. As such, this method is especially suited to the analysis of a large number of cells transiently transfected with a suitable reporter protein. This removes the significant overhead of constructing a stable cell line with fixed copy number for each construct; hence, it facilitates large-scale investigations of transcriptional output.

Although in this study, the algorithm was run on all the cell data at once, the nature of the hierarchical distribution means that the copy number, translation rate and other distributions can be used as a fixed prior in subsequent analysis; hence, comparison between separate runs will still be valid. Also, if experiments were undertaken to investigate the nature of these distributions, the hierarchical model would provide a framework in which this information could be incorporated into the estimation procedure. However, care must be taken to ensure that there is no reason to believe that the distributions will be different in the separate experiments.

Another strength of this hierarchical procedure is that it is inherently flexible and could potentially be used to answer a number of other biological questions, such as how certain stimuli affect the transcription of a gene in a population of cells. The form of the hierarchical distributions can be chosen to fit the investigation, and it would even be possible to incorporate mixtures of distributions or a class allocation methodology if the application warranted it. Furthermore, the model can easily be extended to incorporate oscillatory systems, such as the NF-κB system without requiring a full mathematical model of the entire network (Ashall *et al.*, 2009). This is because the likelihood for each individual cell is fundamentally based on a changepoint model; therefore, we can model oscillations by the addition of more changepoints, similar to the non-hierarchical model in Harper *et al.* (2011).

We applied this method to data gathered from live cell imaging to investigate how the enhancer function of two known *cis*-regulatory elements affects transcription rates in cell populations. Our results confirmed and extended findings based on bulk cell measurements, namely, that the presence of these enhancers leads to increased transcription rates, but we were also able to investigate how each individual cell contributes to the output. Our results indicated a lower fold change than results obtained using bulk cell measurements with the luciferase reporter. However, these experiments are unlikely to be directly comparable, as we use a fluorescent reporter and specifically measure differences in active transcription in our algorithm, whereas the previous test measured luminescence at a single time point regardless of transcriptional activity at that time.

Using our method, we were able to establish that these enhancers do not engender increased transcription rates across all cells, but act to substantially increase transcription rates in a proportion of the population. This implies that transcription of a gene is not always affected by the presence of an enhancer, but those that are affected transcribe at a higher rate. This may be because the transcription factors that interact with an enhancer may not be present or active in every cell; therefore, transcription occurs at a similar level as when the enhancer is not present. Furthermore, by analysing the single-cell estimates we can distinguish between a binary and graded response to the enhancer module and provide a more detailed description of *cis*-regulatory element function. Our data show that both the ProxEnh and DistEnh increase transcription rates in a graded fashion in the responding cells, i.e. in the proportion of cells that have a higher transcription rate than the promoter alone. It will be of interest to test, using a range of enhancers, whether the proportion of responding cells is modulated by enhancer strength. These insights into the nature of transcriptional regulation would be difficult to uncover without recourse to single-cell analysis.

Our hierarchical model enables studies of systems involving intricate transcriptional dynamics and can easily be extended to large-scale investigations by accounting for uncontrolled reporter gene copy numbers inherent in transient transfections. The approach can feasibly be expanded to systematically measure the activity of several hundred *cis*-regulatory element promoter reporter variants in parallel and infer gene regulatory logic. Undertaking very high-throughput studies similar to Melnikov *et al.*, (2012), Patwardhan *et al.* (2012) and Sharon *et al.* (2012) in which potentially several thousands of different gene configurations would be analysed is technically possible with this framework, although the resources needed to automatically segment and track many thousands of individual cells over long time courses would currently impede scaling up to such levels. Also, the computational time required to run the algorithm could be a limiting factor, as the time needed to run the algorithm increases linearly as cell numbers increase, although this could be offset by the use of parallel programming on a suitably large cluster computer. As such, we would recommend that these limitations be taken into account when considering the scope of such a study. However, because of its wide applicability and extensibility, the proposed algorithm provides an invaluable framework for large-scale analysis of enhancer function and the investigation of other transcriptional mechanisms.

## Supplementary Material

Supplementary Data
