# Supplementary material for: A hierarchical model of transcriptional dynamics allows robust estimation of transcription rates in populations of single cells with variable gene copy number
Source: Bioinformatics. 2013 May 14;29(12):1519–25. doi: 10.1093/bioinformatics/btt201 (PMC3673223; doi:10.1093/bioinformatics/btt201)
Supplement: Supplementary Data [file supp_29_12_1519__index.html]

A hierarchical model of transcriptional dynamics allows robust estimation of transcription rates in populations of single cells with variable gene copy number — A hierarchical model of transcriptional dynamics allows robust estimation of transcription rates in populations of single cells with variable gene copy number — Supplementary Data 

# A hierarchical model of transcriptional dynamics allows robust estimation of transcription rates in populations of single cells with variable gene copy number

## Supplementary Data

files

**Files in this Data Supplement:**

- Supplementary Data - ps file
